# Gadolinium-Based Contrast Agents in Pregnant Women: A Literature Review of MRI Safety

**DOI:** 10.7759/cureus.38493

**Published:** 2023-05-03

**Authors:** Sami A Alghamdi

**Affiliations:** 1 Department of Radiological Sciences, College of Applied Medical Sciences, King Saud University, Riyadh, SAU

**Keywords:** safety, foetus, pregnancy, first trimester, gadolinium contrast, mri

## Abstract

Gadolinium-based contrast agents (GBCAs) are commonly used in magnetic resonance imaging (MRI) to enhance the visualisation and characterisation of the region of interest/lesion. Internal structures are well seen with MRI with good spatial resolution. Although MRI is generally considered safe during pregnancy, concerns have been raised regarding the safety of GBCAs, particularly during the first trimester. Limited studies have been conducted to assess the safety of GBCAs in pregnant women, with conflicting results.

A comprehensive literature search was conducted using PubMed, SpringerLink, Medscape, ResearchGate and Wiley Online Library. The search terms included various combinations of MRI, pregnancy, first trimester, gadolinium contrast agents, foetus, risk, and toxicity. The search criteria were articles published in English in the last 20 years and indexed in the MEDLINE or Embase databases.

The majority of studies found no definitive evidence that GBCAs are harmful during pregnancy, particularly during the first trimester. Some studies reported no increased risk of adverse outcomes in infants exposed to GBCAs during the first trimester. However, other studies showed inconsistent results. Retrospective cohort studies provided some reassurance regarding the safety of GBCAs when indicated in pregnant women but did not address potential long-term adverse outcomes in infants exposed to GBCAs during gestation. The literature review also highlights the importance of further evaluating the subacute and chronic effects of GBCA exposure in infants.

The safety of GBCAs during pregnancy, particularly during the first trimester, remains uncertain. More large-scale, long-term studies are needed to clarify the safety of GBCAs in pregnant women and their potential effects on foetal and neonatal outcomes. Until conclusive evidence is available, healthcare providers should carefully weigh the benefits and risks of using GBCAs during pregnancy and consider alternative imaging modalities, such as non-contrast MRI or ultrasound, when necessary.

## Introduction and background

Magnetic resonance imaging (MRI) is a medical imaging technique that makes use of strong magnetic fields that help in producing detailed images of the body. The main role of contrast agents is to improve both the detection and characterisation of lesions. Gadolinium-based contrast agents (GBCAs) are some of the most commonly used agents in MRI to enhance the visibility of blood vessels, organs, and other internal bodies [1]. While they are generally considered safe, there have been concerns about their use during pregnancy, particularly in the first trimester [2]. Therefore, it is important to note that not all MRI exams require a GBCA, and alternatives such as non-contrast MRI or ultrasound may be considered when imaging is necessary during pregnancy.

During the first trimester, the foetus is in a critical development period and exposure to various substances can potentially have harmful effects [3]. However, the available evidence relating to GBCA exposure is not definitive regarding the risks but rather is conflicting [4]. Several studies have investigated the safety of GBCAs in pregnant women, and different outcomes have been reported. For example, a 2018 study published in the journal *Radiology* found no increased risk of adverse outcomes in infants exposed to GBCAs during the first trimester [5]. Another study published in the same journal in 2019 also found no significant differences in outcomes between infants who were exposed to GBCAs and those who were not [6]. To fully comprehend the safety of GBCAs during pregnancy, it is necessary to remember that these studies are constrained by the small sample sizes used. Additionally, each individual situation involving the use of GBCAs during pregnancy needs to be thoroughly examined to weigh the advantages and disadvantages for the mother and the foetus.

Sometimes, foetuses can be unintentionally exposed to gadolinium in the early stages of pregnancy by women who are not yet aware of their pregnancy. It may be possible to decrease unintentional gadolinium contrast doses by paying closer attention to current pregnancy screening procedures [7]. It has not been determined whether GBCAs are safe for pregnant women; hence, their use is not advisable unless necessary for the health of the mother or the foetus [7]. The results of available cohort studies and case reports on the relationship between gadolinium and unfavourable foetal outcomes have been inconsistent.

## Review

Methods

Several methods were utilised for the literature search. The different scientific literature on clinical and preclinical case studies involving the use of MRI and contrast agents for pregnant women at different stages of pregnancy were examined. These studies were found by searching different databases, specifically PubMed, SpringerLink, Medscape, ResearchGate, and Wiley Online library, using the following search terms: ‘MRI AND pregnancy’, ‘first-trimester exposure to gadolinium contrast agents’, ‘MRI contrast agents AND foetus’, ‘risk of foetal and MRI’ and ‘toxicity of MRI contrast agents and pregnancy’. The search criteria were: (a) articles published in the last 20 years; (b) articles published in scientific journals or listed in databases such as MEDLINE or EMBASE; (c) articles published in English; (d) articles covering the search terms and different types of case reviews or studies focusing on the key points of interest.

Results and discussion

MRI Safety During Pregnancy

MRI is generally considered safe for pregnant women. However, there are some important considerations. The main concern regarding MRI during pregnancy is the potential risk to the developing foetus associated with the strong magnetic fields used in the procedure. There is no evidence to suggest that MRI poses any direct risk to the foetus [8].

For the safety of MRI during the first trimester of pregnancy, it is still being studied. For instance, GBCAs are not suggested for use during pregnancy unless necessary for the health of the mother or the foetus. Current research on their safety in pregnant patients is still ongoing, and the findings are mixed [8]. Data from various cohort studies and case reports have revealed inconsistent results regarding the association between GBCAs and poor foetal outcomes [9].

It is important that women inform their healthcare provider about their status before an MRI [10]. Providers need to understand the risk and evaluate if the benefits of MRI outweigh the potential risk to the foetus. MRI is generally considered safe during the second and third trimesters of pregnancy when the risk of harm to the foetus is low. However, some precautions may be necessary, such as using a lower-strength magnetic field.

A retrospective cohort study conducted between 1999 and 2014 that covered more than 11 million pregnancies to evaluate and understand the association between prenatal MRI exposure with or without GBCAs and foetal and neonatal death did not show any high risk but was reassuring regarding the use of gadolinium during pregnancy when indicated [11]. However, the study focused more on evaluating acute effects. Therefore, the amount of contrast agent retained in tissue remains a crucial point of focus for researchers. The impact of contrast agent exposure in terms of subacute and chronic adverse outcomes in infants is also an important focal point that should be further evaluated [11].

Bird et al. [12] investigated the safety of first-trimester exposure to GBCAs during pregnancy, offering a contrasting perspective on MRI safety for pregnant women. The study highlights the difficulty in establishing a definitive risk level due to the differing results in the literature. They also emphasised the importance of using GBCAs during pregnancy only when essential for the health of the mother or the foetus. The researchers analysed a large sample of pregnancies, categorising MRI procedures based on the anatomic location and trimester or by comparing pregnant and matched non-pregnant women [12]. The study found that most pregnant women exposed to contrast-enhanced MRI examinations underwent head MRI. In terms of trimester comparisons, the first trimester had a 4.3-fold higher prevalence of GBCA exposure compared to the second trimester and a 5.1-fold higher prevalence compared to the third trimester. Thus, according to Bird et al. [12], GBCA exposure is more prevalent, albeit inadvertently, during the first trimester compared to the second and third trimesters of pregnancy. A summary of the overall research findings is given in Table 1.

**Table 1:** Summary of research findings GBCAs: gadolinium-based contrast agents.

Study	Cohort size	Timeframe	Findings
Kanda et al. (2016) [8]	N/A	N/A	Mixed results on the safety of GBCAs in pregnant patients
Mervak et al. (2020) [9]	N/A	N/A	Inconsistent results linking GBCAs and poor foetal outcomes
Winterstein et al. (2023) [11]	11 million+	1999–2014	No increased risk of foetal and neonatal death associated with GBCA use but concerns about retention of GBCAs in tissue and subacute/chronic adverse outcomes in infants
Bird et al. (2019) [12]	Large sample	N/A	Difficult to establish definitive risk level. GBCAs should be used during pregnancy only when essential for the health of the mother or foetus. GBCA exposure was higher in the first trimester compared to the second and third trimesters

Short and long-term effects

Understanding the effects of GBCAs depends greatly on understanding the primary clinical recommendations for their use, the dangers for the mother and the foetus, and the side effects. It is crucial to understand that allergic and non-allergic reactions, such as nausea and vomiting, account for most short-term dangers [13]. However, severe reactions that are typical in pregnancy include premature labour, repeated late decelerations of the foetal heart rate, and protracted foetal bradycardia [7]. Retained intracranial gadolinium and nephrogenic systemic fibrosis (NSF) are long-term issues associated with using GBCA MRI [14]. NSF is a severe and uncommon disease that affects people with reduced renal function and is characterised by fibrosing skin lesions and organ failure. No incidences of NSF in a pregnant patient or a newborn following intrauterine exposure have been documented as of yet.

Different studies have contributed to the current understanding of why GBCA is considered to have an increased risk for pregnant women and foetuses [15]. For instance, about 90% of GBCAs are usually found to be eliminated rapidly within 24 hours in individuals with normal renal function, while the remaining 10% takes longer [15]. The long-term risks are more attributable to continuous exposure and accumulation over a long period. In view of such a continuum, unhealthy kidneys create a possibility for increased risk after exposure to GBCA MRI, especially in the first trimester.

In a study by Ray et al. [16], there was an increase in stillbirths and neonatal deaths following exposure to GBCAs used in MRI scans. However, the study found no increased risk of death in 1,720 pregnant women who underwent any type of MRI (both enhanced and unenhanced) during the first trimester. Additionally, the study found a higher incidence of rheumatological, inflammatory, and infiltrative skin conditions among young children who were exposed to GBCA in utero during the first trimester [17]. This heightened risk was only observed during the first trimester, possibly because most enhanced MRIs were performed during this period [18].

The dangers of exposure to GBCAs during pregnancy, particularly during the first trimester, are not well understood. However, some research has indicated that prenatal exposure to GBCAs may be linked to a higher risk of neurodevelopmental problems and other negative effects for the foetus. For instance, in a study published in the *Journal of the American Medical Association*, Ray et al. [2] identified a potential link between GBCA exposure during pregnancy and an increased risk of the child developing a wide spectrum of neurological and kidney diseases. However, further study is required to corroborate these findings because of the study’s limitations, including the small sample size and the retrospective approach.

Given the uncertainties, healthcare providers should meticulously evaluate the potential risks and benefits of GBCA use during pregnancy on an individual basis. Alternative imaging options, such as non-contrast MRI or ultrasound, may be considered when imaging is necessary during pregnancy [19].

Some evidence suggests that exposure to GBCAs during the first trimester may be associated with a slightly increased risk of adverse outcomes, including foetal loss and malformations. However, the overall risk appears to be low, and the benefits of an MRI exam may outweigh the potential risks in some cases. Not all MRI exams require a GBCA, and alternatives such as non-contrast MRI or ultrasound may be considered when imaging is necessary during pregnancy. Additionally, the decision to use a GBCA during pregnancy should be made on a case-by-case basis in consultation with a healthcare provider. In an observational study involving 15 neonates, Jabehdar et al. [20] highlighted the absence of an attributable adverse outcome for a population-based cohort study that involved more than 1.4 million pregnancies. A review of the first trimester with an MRI with or without contrast did not reveal the presence of stillbirths, neonate deaths, congenital anomalies, or neoplasms but did reveal the presence of vision loss in a small group when compared to those that do not undergo an MRI [20].

Toxicity

The risk of toxicity associated with GBCAs during pregnancy is not well understood, especially during the first trimester [21]. However, some studies suggest that exposure to GBCAs during pregnancy may increase the risk of adverse outcomes for both the mother and the developing foetus [10,22].

GBCAs contain gadolinium, a heavy metal that can be toxic in high doses. While the amount of gadolinium in a standard dose of GBCA in MRI is considered safe for most patients, there are concerns that the potential toxicity of gadolinium may be greater during pregnancy due to changes in renal function and the developing foetus’s immature blood-brain barrier. The chelation process after GBCA administration protects the human body from the toxicity of gadolinium and allows its rapid excretion [23]. However, reactions to GBCAs come in various forms, and researchers categorise them as immediate reactions (within one hour of administration), late reactions (within one hour to one week of administration), and very late adverse reactions (within a few days to one month of administration) (NSF) [23]. Chelation enables gadolinium’s quick elimination while shielding the body from its toxicity [23].

Gadolinium and the Periconception Period

The periconception period, which includes the time before conception and the first few weeks of pregnancy, is a critical window for foetal development. Therefore, understanding the effects of gadolinium exposure during this time is crucial to ensuring the health and safety of both the mother and the foetus. De Santis et al. [24] examined the impact of GBCA exposure during the periconceptional period and found no major adverse effects on pregnancy and neonatal outcomes, particularly during the first trimester. However, the sample size in this study was small at only 26 pregnant women, which limits the generalisability of the results. Therefore, further research with larger sample sizes is needed to confirm these findings.

In a larger study, Ray et al. [2] investigated the risk of congenital anomalies and other adverse pregnancy outcomes following gadolinium exposure in the periconception period. They analysed data from 397 pregnancies and found no significant association between GBCA exposure and the risk of major congenital anomalies. However, they observed a small increased risk of stillbirth or neonatal death. These findings suggest that while gadolinium exposure during the periconception period may not be associated with a high risk of congenital anomalies, there may be other potential risks that warrant further investigation.

Gadolinium is known to cross the placenta, and animal studies have demonstrated the potential teratogenic effects of GBCAs on developing foetuses [25]. However, the exact mechanism by which gadolinium could potentially cause harm during pregnancy remains unclear. It is believed that the risk of adverse effects may be related to the specific GBCA used, as well as the dose and timing of exposure. Moreover, the timing of GBCA exposure during pregnancy is an important factor to consider. The first trimester is a critical period for foetal organogenesis, and exposure to potentially harmful agents during this time may have long-lasting effects on foetal development (Table 2) [26]. However, a review by the American College of Radiology (ACR) Committee on Drugs and Contrast Media [19] suggested that there is insufficient evidence to establish a clear association between GBCA exposure during the first trimester and an increased risk of congenital anomalies.

**Table 2:** Factors influencing the risk of adverse effects of gadolinium exposure GBCAs: gadolinium-based contrast agents.

Factor	Potential impact on risk
Type of GBCA	Macrocyclic GBCAs are associated with lower risks compared to linear GBCAs.
Dose and timing	Higher doses and exposure during critical periods (e.g. first trimester) may increase risk.
Individual factors	Patient-specific factors, such as genetic predisposition, may influence the risk of adverse effects.

In light of the potential risks associated with gadolinium exposure during pregnancy, guidelines have been developed to help practitioners make informed decisions regarding the use of GBCAs in pregnant women. The ACR guidelines recommend that GBCAs should only be used during pregnancy if the potential benefits to the mother outweigh the potential risks to the foetus such as pregnant cancer patients [10]. Additionally, if GBCA administration is deemed necessary, the guidelines suggest using the lowest possible dose and considering the use of macrocyclic GBCAs, which have been associated with lower risks compared to linear GBCAs. Frenzel et al. [27] conducted research on rats with normal kidney function and discovered that repeated injections of linear GBCAs result in greater gadolinium retention than macrocyclic compounds.

The current literature on gadolinium exposure during the periconceptional period suggests that there may be no significant association with major congenital anomalies or adverse pregnancy outcomes. However, some studies have indicated a potential increased risk of stillbirth or neonatal death and differences in risk depending on the type of GBCA used. Due to the limitations of existing studies, such as small sample sizes and potential confounding factors, further research is needed to provide a more comprehensive understanding of the safety of GBCAs during the periconceptional period. Future research should aim to address the limitations of previous studies by using larger sample sizes, more diverse populations and prospective study designs. Additionally, investigating the potential long-term effects of GBCA exposure on child development and cognitive function would provide valuable insights into the safety of these agents during pregnancy.

It is also essential to improve our understanding of the mechanisms by which GBCAs may affect foetal development. This may help identify potential biomarkers of GBCA-related adverse effects that could be used to develop safer contrast agents or strategies for mitigating risks associated with their use during pregnancy.

In clinical practice, healthcare providers should carefully weigh the potential benefits and risks of GBCA administration in pregnant women. When possible, alternative imaging modalities, such as ultrasound or non-contrast MRI, should be considered. If GBCA use is deemed necessary, following established guidelines such as those provided by the ACR can help minimise potential risks to both the mother and the foetus.

## Conclusions

According to the findings of the literature reviewed, there is a general consensus that GBCA MRI poses little or no risk to pregnant women. However, there is no conclusion regarding foetal safety. Large cohort studies are not yet available to clarify the risk of neonatal death associated with GBCA use during pregnancy; therefore, more studies on the use of GBCAs in pregnancy and their effects are needed. The safety of MRI with GBCAs for pregnant women remains uncertain.
